# Feasibility and utility of the days of antibiotic spectrum coverage (DASC) in national antimicrobial use surveillance in Japan

**DOI:** 10.1017/ash.2025.10086

**Published:** 2025-08-07

**Authors:** Ichiro Kawamura, Sanae Suzuki, Mio Endo, Masaaki Ogawa, Satoshi Hasegawa

**Affiliations:** 1 Department of Infectious Diseases, Osaka International Cancer Institute, Osaka, Japan; 2 AMR Clinical Reference Center, Japan Institute for Health Security, National Center for Global Health and Medicine, Tokyo, Japan; 3 Pharmacy, Osaka International Cancer Institute, Osaka, Japan

## Abstract

**Objective:**

Days of antibiotic spectrum coverage (DASC) is a novel metric that incorporates the antibiotic spectrum into consumption metrics, addressing the limitations of traditional metrics such as days of therapy (DOT). This study aimed to evaluate the feasibility of integrating DASC into the Japan Surveillance for Infection Prevention and Healthcare Epidemiology (J-SIPHE) system.

**Design:**

Retrospective observational study.

**Setting:**

Hospitals voluntarily participating in J-SIPHE.

**Participants:**

Inpatients from 1,833 hospitals between January 2019 and December 2022.

**Methods:**

Antibiotic use was assessed using DOT, DASC, and DASC/DOT. Antibiotic spectrum coverage scores were assigned based on published data or expert consensus. Annual trends were assessed using median values, and hospital-level variation was explored by hospital size. Proportional use of antibiotic classes by DOT and DASC was compared using 2022 data.

**Results:**

As the number of hospitals participating in J-SIPHE increased over time—particularly small and medium-sized hospitals—median DOT and DASC per 1,000 patient-days declined by 21.2% and 19.1%, respectively, from 2019 to 2022, while DASC/DOT remained stable. In 2022, proportional use of antibiotic classes varied by hospital size, and rankings differed when comparing DOT- and DASC-based measures. Broad-spectrum agents such as carbapenems and fluoroquinolones ranked higher by DASC than DOT. Hospital-level distributions of DOT and DASC/DOT showed substantial variation across hospital sizes.

**Conclusions:**

Integration of DASC metrics into national surveillance is feasible. DASC and DASC/DOT complement DOT by incorporating spectrum breadth, providing more comprehensive insight into antimicrobial use patterns and supporting stewardship benchmarking and intervention planning.

## Introduction

Monitoring antimicrobial use in healthcare facilities is a critical component of evaluating antimicrobial stewardship activities. Among the various metrics available to quantify antibiotic consumption, days of therapy (DOT) is one of the most widely utilized metrics globally.^1,2^ However, this conventional metric does not account for the breadth of the antibiotic spectrum, limiting its ability to reflect stewardship efforts aimed at minimizing the unnecessary use of broad-spectrum antibiotics, particularly in empirical and de-escalation settings.^3^ To address this limitation, a novel metric—days of antibiotic spectrum coverage (DASC)—was introduced in 2022. DASC serves as a composite metric that captures not only the quantity of antibiotic therapy but also the breadth of spectrum exposure.^4^

This concept builds on earlier efforts to quantify spectrum breadth, most notably the Antibiotic Spectrum Index (ASI) developed by Gerber et al and the modified ASI by Ilges et al.^5,6^ While these indices have been valuable for comparing the relative spectrum of individual agents, they were not originally designed for aggregate, hospital-level surveillance. DASC expands on these frameworks by incorporating antibiotic spectrum scores into a DOT-based metric, allowing for scalable, population-level monitoring of antibiotic spectrum use.

Initially developed for hospital-level surveillance, the DASC methodology also holds promise for integration into national antimicrobial use surveillance systems. One of the key objectives of hospital-level antimicrobial use surveillance at the national level is to provide benchmark data that can support local antimicrobial stewardship initiatives, inform public health strategies, guide research activities, and shape national policies aimed at optimizing antimicrobial use and addressing antimicrobial resistance.^7^ While DOT has been adopted as a standard metric for national antibiotic utilization in countries such as the United States and Japan, DASC has yet to be incorporated into such frameworks.^2,8^

Leveraging existing national surveillance infrastructure represents a strategic and pragmatic approach for incorporating DASC. However, the successful implementation of DASC in national surveillance requires the development of a robust surveillance framework and standardized operational protocols. In Japan, the Japan Surveillance for Infection Prevention and Healthcare Epidemiology (J-SIPHE) functions as the national platform for monitoring antimicrobial use and routinely collects DOT-based data. This study aims to evaluate the feasibility of integrating DASC and its related metrics into the national antimicrobial use surveillance system and to examine the associated challenges and practical considerations for implementation.

## Methods

### Study setting

A retrospective observational study was conducted using data from the J-SIPHE inpatient database, covering the period from January 2019 to December 2022. The J-SIPHE system was launched in 2019 following the National Action Plan on Antimicrobial Resistance formulated by the Japanese government.^9^ This national database contains comprehensive information on hospitals, including basic institutional characteristics, antimicrobial use data, and details related to antimicrobial stewardship teams. As of 2022, approximately 2,000 hospitals were enrolled in J-SIPHE, consisting almost entirely of acute-care institutions.

For all inpatients at each hospital, the following data were collected: (1) patient-days for inpatients, (2) DOT for each antibiotic agent, and (3) number of hospital beds. Hospitals were categorized into three groups based on bed capacity: large hospitals (500 or more beds), medium-sized hospitals (100 – 499 beds), and small hospitals (20 – 99 beds).^10^ In Japan, medical facilities with 20 or more beds are officially classified as hospitals.

### Selection of antibiotics and definition of ASC score

This study included all intravenous and oral antibiotics registered in the J-SIPHE system. The following agents were excluded from the analysis: those used for the treatment of tuberculosis (eg, kanamycin and streptomycin), syphilis (eg, benzathine penicillin), gonorrhea (eg, spectinomycin), *Clostridioides difficile* infections (eg, fidaxomicin and oral vancomycin), congenital toxoplasmosis (eg, spiramycin), and selective digestive decontamination (eg, kanamycin, oral colistin, and oral polymyxin B). Additionally, antifungal and antiviral agents were excluded, as these were not covered in previous DASC-related studies.^3,4,11,12^

Antibiotic agents were classified according to the Anatomical Therapeutic Chemical (ATC) classification system, following the ATC/DDD Index 2025.^13^ The antibiotic spectrum coverage (ASC) scores used to calculate DASC were based on previously published studies.^3,4,12–14^ For antibiotics assigned different ASC scores in multiple studies and those without an established ASC score in the literature, scores were determined through discussions among antimicrobial stewardship program (ASP) clinical pharmacists (S.S., M.E., M.O., and S.H.) and an infectious disease physician (I.K.). Table 1 provides the ASC scores for the antibiotics included in this study, and the rationale behind these scores is detailed in Supplementary Table 1.

**Table 1.** Antibiotic spectrum coverage (ASC) scores for 88 antibiotics

ASC Score	Antibiotic agents
1	None
2	Metronidazole
3	Benzylpenicillin, cefalotin, cefazolin, cefalexin, cefroxadine, cefixime, ceftibuten, erythromycin
4	Cefotiam, cefuroxime, cefaclor, cefpodoxime, cefdinir, cefteram, aztreonam
5	Ampicillin, amoxicillin, bacampicillin, cefminox, cefmenoxime, cefditoren, cefcapene, clarithromycin, quinupristin/dalfopristin, vancomycin, teicoplanin
6	Ampicillin/cloxacillin, cefotaxime, ceftazidime, ceftriaxone, latamoxef, azithromycin, roxithromycin, clindamycin, lincomycin, colistin, linezolid, daptomycin, tedizolid
7	Demethylchlortetracycline, doxycycline, tetracycline, piperacillin, amoxicillin/clavulanate, cefmetazole, flomoxef, cefozopran, sulfamethoxazole/trimethoprim, josamycin, dibekacin, arbekacin
8	Minocycline, ampicillin/sulbactam, sultamicillin, cefepime, cefpirome, ceftolozane/tazobactam, isepamicin, lomefloxacin, fosfomycin
9	Chloramphenicol, cefoperazone/sulbactam, tebipenem, faropenem, tobramycin, gentamicin, amikacin, ciprofloxacin, ofloxacin, ciprofloxacin, norfloxacin
10	None
11	Piperacillin/tazobactam, pazufloxacin
12	Meropenem, doripenem, biapenem, imipenem/cilastatin, panipenem/betamipron, levofloxacin, prulifloxacin
13	Imipenem/cilastatin/relebactam, lascufloxacin, moxifloxacin, garenoxacin
14	Tosufloxacin
15	Tigecycline
16	Sitafloxacin

ASC score, Antibiotic Spectrum Coverage score.

### Evaluation of antibiotic use by DOT, DASC, and DASC/DOT

DOT was defined as the aggregate number of days for which a patient received a specific antibiotic, regardless of dose. Patient-days were calculated as the total number of days all patients spent in the hospital during the reporting period. Unlike the Antimicrobial Use and Resistance Module of the National Healthcare Safety Network in the United States, which uses days present as the denominator, the J-SIPHE system adopts the conventional patient-days metric for calculating antimicrobial use.^15^

DOT was expressed per 1,000 patient-days and served as a consumption metric, representing the amount of antibiotic therapy administered to inpatients. DASC was calculated by multiplying DOT by the ASC score assigned to each antibiotic. DASC was also expressed per 1,000 patient-days, serving as a composite metric that reflects both the quantity and spectrum of antibiotic use. Additionally, the DASC/DOT ratio was further calculated to represent the average antibiotic spectrum per patient per therapy day.

Annual trends were evaluated using median values, and interquartile ranges (IQRs) were also calculated to describe variability. Variations at the hospital level were assessed by hospital size. The proportional use of antibiotic classes, based on both DOT and DASC, was compared using 2022 data.

## Results

### Characteristics of participating hospitals

**Figure 1.**
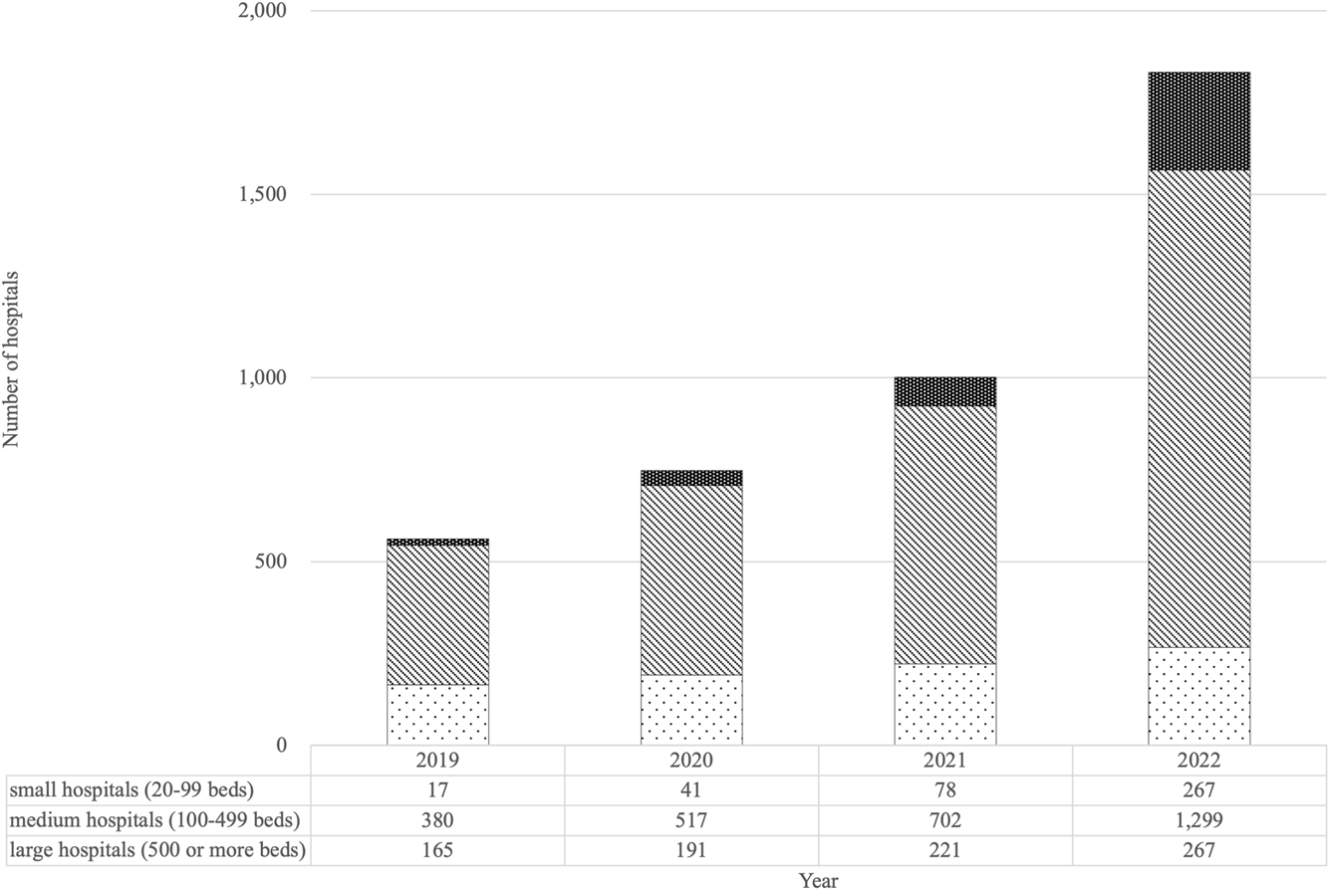
Cumulative number of hospitals enrolled in the J-SIPHE (2019 – 2022).

A total of 1,833 hospitals were included in the analysis, comprising 267 small hospitals (20 – 99 beds), 1,299 medium-sized hospitals (100 – 499 beds), and 267 large hospitals (500 or more beds). The number of facilities participating in the J-SIPHE increased annually from 562 in 2019 to 1,833 in 2022 (Figure 1). Given that Japan has approximately 8,000 hospitals nationwide, the participating facilities represent about one-quarter of all hospitals in the country. Regarding hospital size distribution, large hospitals accounted for 29.4%, medium-sized hospitals for 67.6%, and small hospitals for 3.0% in 2019. By 2022, the proportion of large hospitals had decreased to 14.6%, while medium-sized and small hospitals accounted for 70.9% and 14.6%, respectively.

### National trends in DOT, DASC, and DASC/DOT

Table 2 summarizes national trends in DOT, DASC, and DASC/DOT from 2019 to 2022 across all hospitals participating in the J-SIPHE, presented as medians and IQRs. Among all hospitals, both DOT and DASC per 1,000 patient-days demonstrated a consistent decline over time, while DASC/DOT remained unchanged. Specifically, the median DOT decreased by 21.2%, from 388.2 in 2019 to 305.9 in 2022. Similarly, the median DASC declined by 19.1%, from 2,643.4 to 2,138.4. In contrast, the median DASC/DOT remained stable at 6.95 in both 2019 and 2022. When stratified by hospital size, DOT and DASC values remained relatively stable in large hospitals, whereas small and medium-sized hospitals exhibited more marked fluctuations over the study period.

**Table 2.** Annual trends in DOT, DASC, and DASC/DOT among hospitals participating in J-SIPHE

	2019	2020	2021	2022
DOT/1,000 patient-days				
all hospitals	388.2 (280.9 – 463.6)	382.4 (263.8 – 480.4)	360.7 (240.7 – 471.4)	305.9 (176.9 – 436.0)
large hospitals	472.9 (423.2 – 537.9)	492.9 (428.6 – 553.9)	502.2 (440.5 – 564.0)	489.3 (435.6 – 559.9)
medium hospitals	350.8 (261.6 – 421.4)	341.7 (249.1 – 433.4)	331.3 (227.1 – 424.7)	296.8 (177.4 – 407.2)
small hospitals	202.4 (152.7 – 272.8)	222.0 (146.6 – 302.2)	207.0 (137.9 – 296.1)	183.0 (116.7 – 266.1)
DASC/1,000 patient-days				
all hospitals	2,643.4 (1,979.7 – 3,211.7)	2,580.1 (1,844.9 – 3,177.9)	2,491.2 (1,668.2 – 3,164.0)	2,138.4 (1,242.7 – 2,972.8)
large hospitals	3,153.2 (2,801.5 – 3,583.2)	3,165.0 (2,809.6 – 3,591.0)	3,258.7 (2880.7 – 3 607.5)	3,189.1 (2,859.6 – 3,636.3)
medium hospitals	2,450.9 (1,834.8 – 2,970.4)	2,350.4 (1,682.6 – 2,982.1)	2,282.3 (1,564.9 – 2,937.4)	2,078.1 (1,260.2 – 2,839.3)
small hospitals	1,360.5 (1,125.2 – 1,942.6)	1,388.7 (1,002.4 – 2,043.9)	1,457.8 (1,018.1 – 2,217.7)	1,319.8 (836.5 – 1,937.9)
DASC/DOT				
all hospitals	6.95 (6.58 – 7.32)	6.82 (6.46 – 7.19)	6.80 (6.44 – 7.20)	6.95 (6.55 – 7.39)
large hospitals	6.66 (6.40 – 7.09)	6.56 (6.30 – 6.82)	6.49 (6.25 – 6.79)	6.55 (6.29 – 6.80)
medium hospitals	7.06 (6.70 – 7.41)	6.94 (6.56 – 7.27)	6.90 (6.55 – 7.28)	7.01 (6.64 – 7.44)
small hospitals	7.12 (6.68 – 7.70)	7.09 (6.50 – 7.45)	7.04 (6.37 – 7.60)	7.09 (6.63 – 7.73)

Data are presented as median (interquartile range).

DOT, days of therapy; DASC, days of antibiotic spectrum coverage

### Comparison of proportionate use of antibiotic classes by DOT and DASC across hospital sizes in 2022

Table 3 presents a comparison of the proportionate use of antibiotic classes based on DOT and DASC in 2022, stratified by hospital size. Across all hospital sizes, combinations of penicillins (including those with beta-lactamase inhibitors) accounted for the largest proportion of use in both metrics, except for DOT in small hospitals. Notably, third-generation cephalosporins showed the most pronounced variation across hospital sizes. Their DOT-based proportions were 9.8% in large hospitals, 15.4% in medium-sized hospitals, and 21.5% in small hospitals, while the corresponding DASC-based proportions were slightly lower at 8.7%, 14.7%, and 20.1%, respectively. In contrast, combinations of sulfonamides and trimethoprim exhibited the opposite trend, with usage proportionally decreasing from large to small hospitals: the DOT-based proportions were 15.5%, 10.5%, and 7.1%, and the DASC-based proportions were 15.6%, 11.0%, and 7.4%, respectively.

**Table 3.** Comparison of proportionate use of antibiotic classes by DOT and DASC across hospital sizes (2022)

	DOT (%) (Rank)				DASC (%) (Rank)			
Antibiotic classes	All	Large	Medium	Small	All	Large	Medium	Small
Tetracyclines	2.8 (11)	2.3 (12)	3.2 (10)	4.1 (10)	3.3 (9)	2.6 (10)	3.8 (8)	4.9 (8)
Amphenicols	.0 (21)	.0 (21)	.0 (21)	.0 (21)	.0 (21)	.0 (21)	.0 (21)	.0 (21)
Penicillins with extended spectrum	4.6 (9)	5.0 (8)	4.2 (9)	4.2 (9)	3.5 (8)	3.7 (8)	3.4 (10)	3.6 (9)
Beta-lactamase sensitive penicillins	.1 (18)	.1 (18)	.1 (18)	.0 (18)	.0 (18)	.0 (18)	.0 (18)	.0 (18)
Combinations of penicillins, incl. beta-lactamase inhibitors	18.8 (1)	18.5 (1)	19.0 (1)	17.6 (2)	24.9 (1)	23.8 (1)	25.7 (1)	24.2 (1)
First-generation cephalosporins	11.3 (4)	12.2 (3)	10.7 (3)	7.8 (6)	5.0 (7)	5.2 (7)	4.8 (7)	3.5 (10)
Second-generation cephalosporins	8.6 (6)	8.2 (6)	9.0 (5)	8.2 (4)	7.8 (6)	7.1 (6)	8.3 (6)	6.7 (6)
Third-generation cephalosporins	13.0 (2)	9.8 (4)	15.4 (2)	21.5 (1)	12.1 (4)	8.7 (5)	14.7 (3)	20.1 (2)
Forth-generation cephalosporins	1.9 (12)	2.5 (11)	1.4 (12)	1.3 (12)	2.3 (11)	2.9 (9)	1.7 (11)	1.6 (11)
Monobactams	.0 (19)	.0 (19)	.0 (19)	.0 (19)	.0 (19)	.0 (19)	.0 (19)	.0 (19)
Carbapenems	5.8 (7)	5.7 (7)	5.9 (7)	5.9 (8)	10.3 (5)	9.7 (4)	10.8 (5)	10.5 (4)
Other cephalosporins and penems^a^	.2 (17)	.1 (17)	.2 (17)	.1 (17)	.2 (17)	.2 (17)	.2 (17)	.1 (17)
Combinations of sulfonamides and trimethoprim	12.7 (3)	15.5 (2)	10.5 (4)	7.1 (7)	13.2 (3)	15.6 (2)	11.0 (4)	7.4 (5)
Macrolides	4.8 (8)	3.7 (9)	5.5 (8)	7.9 (5)	3.2 (10)	2.4 (12)	3.8 (9)	5.1 (7)
Lincosamides	1.1 (13)	1.1 (13)	1.0 (13)	.9 (15)	.9 (14)	.9 (15)	.9 (14)	.8 (15)
Streptogramins	.0 (22)	.0 (22)	.0 (22)	.0 (21)	.0 (22)	.0 (22)	.0 (22)	.0 (21)
Other aminoglycosides^b^	.9 (16)	.9 (16)	.9 (15)	1.0 (13)	1.2 (13)	1.1 (13)	1.2 (13)	1.3 (13)
Fluoroquinolones	8.8 (5)	8.8 (5)	8.8 (6)	8.8 (3)	15.6 (2)	15.0 (3)	15.8 (2)	15.8 (3)
Glycopeptide antibacterials	2.8 (10)	3.5 (10)	2.3 (11)	2.0 (11)	2.1 (12)	2.5 (11)	1.7 (12)	1.5 (12)
Polymyxins	.0 (20)	.0 (20)	.0 (20)	.0 (20)	.0 (20)	.0 (20)	.0 (20)	.0 (20)
Imidazole derivatives and nitroimidazole derivatives	1.0 (14)	1.0 (14)	.9 (14)	.7 (16)	.3 (16)	.3 (16)	.3 (16)	.2 (16)
Other antibacterials^c^	.9 (15)	1.0 (15)	.8 (16)	.9 (14)	.9 (15)	.9 (14)	.9 (15)	1.0 (14)

DASC, days of antibiotic coverage; incl., including

a Other cephalosporins and penems included the following antibiotic agents: ceftolozane/tazobactam and faropenem.

b Other aminoglycosides included the following antibiotic agents: tobramycin, gentamicin, amikacin, dibekacin, isepamicin, and arbekacin.

c Other antibacterials included the following antibiotic agents: fosfomycin, linezolid, daptomycin, and tedizolid.

Several major antibiotic classes exhibited notable shifts in ranking when comparing DOT-based and DASC-based proportions. For example, across all hospitals, first-generation cephalosporins ranked fourth by DOT proportion but fell to seventh by DASC proportion; carbapenems rose from seventh (DOT) to fifth (DASC); and fluoroquinolones increased from fifth (DOT) to second (DASC). Similar trends were observed across hospital size categories.

### Distribution of DOT and DASC/DOT by hospital size

**Figure 2.**
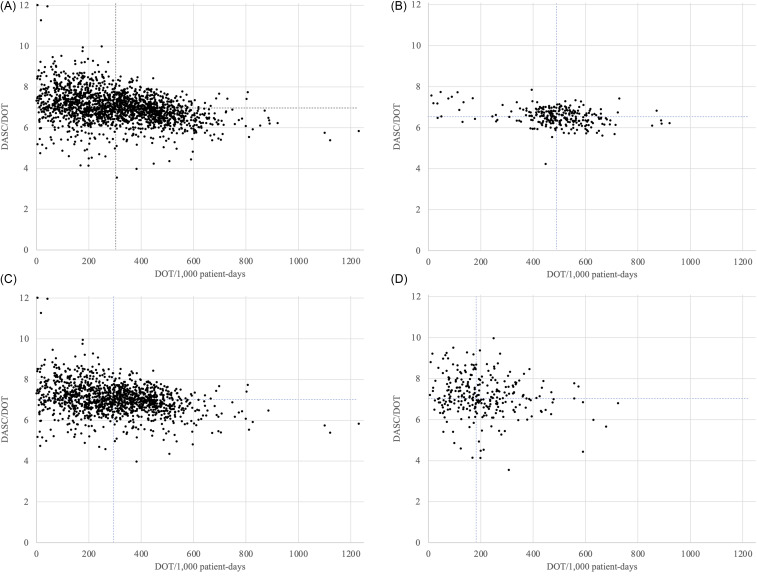
Distributions of DOT/1,000 patient-days and DASC/DOT in 2022. A: in all hospitals; B: in large hospitals; C: in medium-sized hospitals; D: in small hospitals. Vertical and horizontal dot lines represent the median value of each axis. DOT, days of therapy; DASC, days of antibiotic spectrum coverage.

Figure 2 displays the distributions of DOT/1,000 patient-days and DASC/DOT across all hospitals in 2022, categorized by hospital size. The overall median values were 305.9 for DOT and 6.95 for DASC/DOT (Figure 2A). Stratified by hospital size, large hospitals showed median values of 489.3 for DOT and 6.55 for DASC/DOT (Figure 2B), medium-sized hospitals 296.8 and 7.01 (Figure 2C), and small hospitals 183.0 and 7.09, respectively (Figure 2D).

## Discussion

This study assessed temporal trends in inpatient antibiotic use across hospitals participating in the J-SIPHE system from 2019 to 2022, using DOT, DASC, and DASC/DOT as key metrics. When evaluated using median values, both DOT and DASC per 1,000 patient-days showed consistent declines over the four-year period, while DASC/DOT remained stable. These national trends may have been partially influenced by the increasing participation of small and medium-sized hospitals in the surveillance system. Compared to large hospitals, small and medium-sized hospitals are more likely to care for patients with less severe conditions or provide subacute and rehabilitative care, which may result in lower overall antimicrobial exposure, as reflected in both DOT and DASC values. As the expansion of facility enrollment stabilizes, these values are expected to become more reflective of true national trends. In this context, the 2022 data may represent the most reliable estimates of antimicrobial use at the national level to date.

The variation in proportional use of specific antibiotic classes across hospital sizes highlights differences in prescribing patterns that may reflect institutional characteristics such as available stewardship resources, diagnostic capacity, and patient population.^16^ The utility of DASC lies in its ability to capture not only the quantity but also the breadth of antibiotic exposure, thereby offering a more comprehensive assessment than DOT alone. In our study, this is evident in the case of large hospitals, where carbapenems rank seventh based on DOT proportion but rise to fourth when evaluated by DASC, while first-generation cephalosporins rank third by DOT but fall to seventh by DASC. This discrepancy illustrates how DASC provides a more spectrum-sensitive evaluation of antibiotic use compared to DOT.

The observed shifts in ranking of major antibiotic classes between DOT-based and DASC-based proportions underscore the added value of spectrum-adjusted metrics in antibiotic surveillance. Although fluoroquinolones and carbapenems represent a relatively small share of DOT, their broad-spectrum nature causes them to contribute significantly more to overall spectrum exposure, as reflected by their higher DASC rankings. These agents are recognized drivers of antibiotic resistance, and their use warrants close monitoring.^17,18^ Conversely, narrower-spectrum agents like first-generation cephalosporins appear to have less impact on spectrum burden, as indicated by their lower DASC-based rankings. These differences support the use of DASC alongside traditional DOT metrics, particularly for benchmarking antibiotic use, guiding stewardship interventions, and assessing the potential selection pressure associated with broad-spectrum agents.

Importantly, it is also meaningful to deconstruct DASC into its components—DOT and DASC/DOT—for independent evaluation. As previously established, DOT/1,000 patient-days and DASC/DOT are distinct and non-correlated metrics, each capturing different aspects of antibiotic use.^3,4,19^ From a national surveillance perspective, establishing stable baselines for both metrics would enable longitudinal monitoring and support strategic interpretation of stewardship priorities. Because DOT and DASC/DOT capture distinct dimensions of antibiotic use, identifying which metric deviates more from its baseline can help determine whether interventions should focus on reducing overall antibiotic consumption or shifting toward narrower-spectrum agents through de-escalation.

This type of visualization may also be leveraged for feedback-driven antimicrobial stewardship. For instance, hospitals located in the upper right quadrant of each panel—indicating both high DOT and high DASC/DOT—could be identified as candidates for targeted stewardship interventions. In contrast, hospitals with low values for both metrics may serve as benchmarks of optimal practice. These patterns suggest that plotting DOT and DASC/DOT simultaneously can provide intuitive, hospital-level insights and may be adapted into audit-and-feedback tools such as report cards or dashboards. Future efforts could explore how such visual tools might inform benchmarking strategies and promote rational antibiotic prescribing across hospital types.

To date, the only other national surveillance study employing the DASC metric is that of Kanda et al. (2024), which used the Diagnosis Procedure Combination (DPC) inpatient database to assess trends across 634 Japanese hospitals from 2014 to 2018.^3^ While direct comparisons are limited due to differences in hospital populations, ASC score definitions, and antibiotic selection, the median values in our 2022 data set were relatively similar to those reported by Kanda et al for the year 2018: DOT per 1,000 patient-days (305.9 vs 319), DASC per 1,000 patient-days (2,138.4 vs 2,283), and DASC/DOT (6.95 vs 7.21), respectively. Taken together, these results provide a reference point for national antimicrobial use, with DOT around 310, DASC around 2,200, and DASC/DOT around 7, while acknowledging limitations in cross-study comparability.

The methodology for the DASC metric is designed for the continuous surveillance of antibiotic consumption at the hospital level but may also be applicable to subnational, national, and supranational surveillance systems. Aggregating and analyzing data across hospitals can provide valuable insights into the consumption and spectrum of hospital antibiotic agents within a country. From a national surveillance perspective, an ideal trend would be a gradual decline in DOT and DASC/DOT over time, ultimately leading to a corresponding decrease in DASC when assessed collectively. While the current study reflects early trends within an expanding and increasingly diverse hospital cohort, future surveillance with a more stable and comprehensive hospital base will enable more accurate monitoring of longitudinal trends in DOT and DASC/DOT.

Several practical considerations must be addressed before DASC and DASC/DOT metrics can be effectively implemented in national surveillance systems. One important issue is the inconsistency or absence of ASC scores for certain antibiotics, which necessitates standardization through expert consensus. For instance, cefoperazone/sulbactam has been assigned ASC scores ranging from 9 to 11 across studies.^3,12,14^ In Japan, Maeda et al addressed this challenge by assigning ASC scores to a number of antibiotics previously lacking such definitions.^14^ Another consideration is the need to exclude agents outside the scope of DASC. In this study, we excluded antituberculosis agents, syphilis treatments, gonorrhea treatments, *Clostridioides difficile* therapies, congenital toxoplasmosis prophylaxis, and gastrointestinal decontamination agents. Nevertheless, some agents such as kanamycin, which have multiple indications, require careful consideration regarding their classification and primary use within national practice.

This study has several limitations. First, participation in the J-SIPHE is voluntary and consists primarily of acute-care hospitals, which may not be representative of all healthcare facilities in Japan, introducing potential selection bias and limiting generalizability. In addition, the number of participating small and medium-sized hospitals increased substantially over the study period, which may have contributed to the greater year-to-year variability observed in these hospital categories. However, it remains uncertain whether this explains the observed fluctuations entirely, and further investigation is warranted to clarify the underlying causes. Second, although ASC scores were primarily derived from prior literature, inconsistent or unavailable values for certain antibiotics required expert consensus, which may introduce subjective bias and affect reproducibility. Third, DASC and DASC/DOT calculations were based on administrative data without clinical context; thus, the appropriateness of antibiotic use could not be fully evaluated. Fourth, certain antibiotics included in the ASC or available in Japan may be unapproved or rarely used in other countries, which could limit the international applicability of our findings and hinder cross-national comparisons of DASC-based metrics.

In conclusion, this study demonstrates the feasibility of integrating DASC and DASC/DOT into national antimicrobial surveillance systems using existing infrastructure. These metrics offer a more comprehensive understanding of antibiotic use and can inform targeted stewardship interventions nationally.

## Supporting information

10.1017/ash.2025.10086.sm001 Kawamura et al. supplementary material Kawamura et al. supplementary material

## Supplementary material

The supplementary material for this article can be found at https://doi.org/10.1017/ash.2025.10086.
